# The Use of Precision Medicine to Support the Precision of Clinical Decisions in care delivery

**DOI:** 10.1055/s-0044-1800738

**Published:** 2025-04-08

**Authors:** Lina Sulieman, Allison B. McCoy, Lipika Sama, Josh F. Peterson

**Affiliations:** 1 Department of Biomedical Informatics, Vanderbilt University Medical Center, Nashville, TN; 2 Department of General Internal Medicine, Harvard Medical School, Boston, MA

**Keywords:** Precision medicine, clinical decision support, pharmacogenomics, social determinants of health, disease risk, genomics

## Abstract

**Objectives** : Objective: Precision medicine uses individualized patient data, including genomic and social determinants of health SDoH), to provide optimized personalized patient treatment. In this scoping review, we summarize studies published in the last two years that reported on implementation of precision medicine in clinical decision support (CDS) related to precision medicine.

**Methods** : We searched PubMed for manuscripts published in 2022 and 2023 to retrieve publications that included CDS and precision medicine keywords and Mesh terms. We reviewed the abstracts and full texts to apply the inclusion criteria that the study must have described the implementation of precision medicine related CDS within electronic health records. We extracted the domain, type of data used in CDS, target population included in the implementation from the final set of included manuscripts.

**Results** : Our search retrieved 285 manuscripts and papers. Sixteen (16) papers met inclusion criteria after manual review of the full text. Eight of the reviewed papers studied the successful implementation of pharmacogenomics in CDS, four studies investigated the implementation of disease risk, and only one paper described the implementation of CDS integrating social determinants of health.

**Conclusion** : Our scoping review of recent literature highlighted several findings. Pharmacogenomics is the most implemented precision medicine intervention based on published studies. Few reports describing disease risk and polygenic risk scores were found and no study addressed CDS for continuous biometric monitoring. Despite the increasing attention to social determinants of health as a key predictor of health outcomes, only one CDS incorporating SDoH have been publicly reported. Regular updates to scoping reviews can investigate barriers to implementation and identify solutions.

## 1. Introduction

Precision medicine is the personalized approach to clinical care that tailors disease prevention and treatment by considering individual patient characteristics, such as genetics, environment, and lifestyle. Making those data available at the point of care can improve the accuracy of disease diagnosis, identify individuals at increased risk of disease, and improve patient outcomes through earlier diagnosis and better targeted treatment [ 1 , 2 ]. Precision medicine was introduced to refine and complement the dominant model of medical care, evidence-based medicine, which has been critiqued as an all purpose “one-size-fits-all” approach [ 3 ].

Clinical Decision Support (CDS) systems enhance the decision-making process by ideally providing the right information, to the right person, through the right channel, in the right format, and at the right time in the workflow [ 4 , 5 ]. CDS systems attain patients' information from Electronic Health Records (EHR) and utilize computerized clinical knowledge to provide evidence-based suggestions. Precise CDS systems may be able to harness multi-omics data to provide individualized treatment and diagnoses based on clinical and published knowledge [ 6 ]. CDS has been used to suggest differential diagnoses, manage disease risk, advise on treatment strategies, and improve drug safety [ 6 , 7 ]. CDS systems have multiple functionalities, including alert systems, recommendations, disease management, medication prescription and management, and computerized guidelines [ 6 ].

Significant breakthroughs in precision medicine, especially in the availability, understanding, and clinical utility of genomics, have increased the necessity for developing CDS. Efficient and successful implementation of precision medicine CDS tools demonstrates its value in providing recommendations. As an example, one type of CDS can significantly affect healthcare providers' use of pharmacogenomic testing [ 8 ]. Pharmacogenomic Resource for Enhanced Decisions in Care and Treatment (PREDICT), a pharmacogenomics initiative, implemented drug-gene interactions alert systems [ 9 , 10 ]. For example, PREDICT alerts clinicians if they prescribe clopidogrel to patients with homozygous CYP2C19*2 variants to avoid ineffective antiplatelet treatment that may lead to increased cardiovascular event rates.

Traditionally, precision medicine, especially in its implementation, is focused on genetics. More recently, other domains of precision medicine, social determinants of health (SDoH), sensor data, and environmental data have been recognized to play a significant role in creating individualized treatment plans tailored to patients' needs [ 11 , 12 ]. However, despite the abundance of research, implementation of precision medicine using CDS has been limited [ 13 ]. Regularly assessing the status and trends of implementing precision medicine in CDS is vital to understanding the progress and challenges in this area. The previous precision medicine reviews focused on CDS involving genomic medicine and reviewed the literature through March 2023 [ 14 , 15 ].

In this scoping review, we sought to: (i) update prior reviews by summarizing studies published in 2022 and 2023, (ii) determine whether CDS within genomic medicine was expanding in scope to involve sequencing and disease risk scenarios, and (iii) determine whether precision medicine CDS has expanded to include environmental data, SDoH, or models of disease risk that integrate genetic, clinical, and environmental risks. To be included in this scoping review, the CDS must have been implemented and published within the most recent two calendar years, spanning publication dates in 2022 and 2023.

## 2. Methods

From PubMed, we extracted papers that were published in 2022 and 2023. We included papers written in English, which had full text available. The abstract had to include any of the following CDS keywords: (“cognitive aid,” “user interface,” “expert system,” “decision support systems,” “CDS,” “clinical reminder,” “best practice advisory,” “best practice alert,” “decision support,” “Decision Support Systems, Clinical”, and “Decision Support Systems, Management”), and any precision medicine keywords: (“Precision medicine”, “personalized medicine”, “Genomic medicine”, “genomics”, “Pharmacogenomics”, “Pharmacogenetics”, “disease risk”, “somatic variation”, “SDoH”, “Social determinants of health”, “activity sensor”, “home sensor”, “personalized medicine”, “genetics”, “Individualized medicine”, “P4 medicine”, “polygenic risk score”, “genetic variant”, “Biomarker”, “Precision diagnostics”,” Socioeconomic status”, “Health literacy”). We reviewed the abstracts and included the papers that discussed using precision medicine CDS.

Afterward, we read the full-text papers and applied the two inclusion criteria:

- Leveraging patient-specific information, including biomarkers, genes, and social factors;
- EHR implementation: CDS tools had to be described and implemented within an EHR environment. In addition, the paper should describe an applied CDS study that included patients as part of implementation to exclude papers that describe only the framework.

For the papers included in the final dataset, we extracted the following information: precision medicine aspect, the population included in the implementation, CDS type, and a brief description of the CDS implementation.

## 3. Results

**Figure 1.**
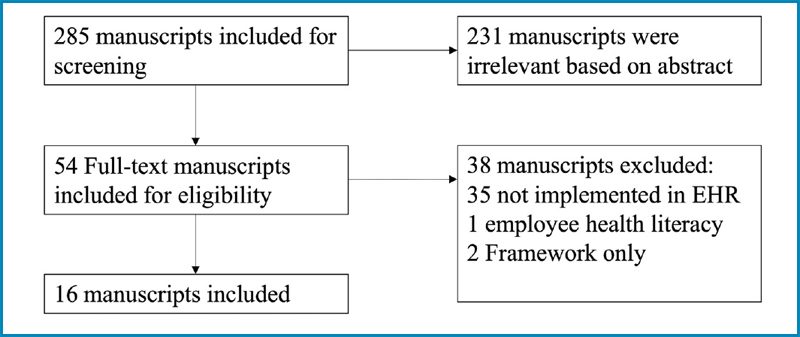
Screening results for manuscripts.

Our PubMed search yielded 285 papers. After reviewing the abstracts, we included 54 papers for full-text review ( Figure 1 ). Ultimately, only 16 papers met the inclusion criteria ( Table 1 ). We identified six precision medicine aspects: pharmacogenomics, genetic screening, Polygenic risk score (PRS), disease risk, SDoH, and other clinical implementations such as referrals. The most implemented precision medicine domain was pharmacogenomics (n=8). The second most implemented precision medicine domain was genetic screening (n=4). The most screened gene variants were CYP2C19 (n=3), SLCO1B1 (n=3), CYP2C9 (n=2), CYP3A4 (n=2), and OPRM1 (n=2). Two studies did not mention variants, medications, or disease and focused on overall implementation.

**Table 1.** Summary of Precision Medicine CDS Studies. PGx: pharmacogenomics, PRS: Polygenic risk score, CDS: Clinical decision support, SDoH: Social Determinant of Health

Title	PM aspect	Patient population	CDS type	CDS implementation	Outcome
Real-World Impact of a Pharmacogenomics-Enriched Comprehensive Medication Management Program [ 16 ]	PGx	Older patients >=65 (28,619 participants: 4,716 intervention and 22,357 in control, 572 declined participation in program)	Medication management system	CDS for pharmacists to create, plan, and propose useful changes medications to be added, removed, modified, monitored, and be alerted for potential future issues	Lowering medication risk (drug-drug interaction), lowering health care utilization
Personalized medicine in a community health system: the NorthShore experience [ 25 ]	PGx, PRS, Genetic screening	35,432 patients	Ordering system, return of genetic results system	Implementation of personalized medicine program with genetic screening, pharmacogenomics screening, and PRS for personalized screening	Hospital admissions, readmissions, and analyses of the relationship between multimorbidity, polypharmacy, social determinants of health, and gene–drug interactions
Pilot Findings of Pharmacogenomics in Perioperative Care: Initial Results From the First Phase of the ImPreSS Trial [ 17 ]	PGx	71 patients, 166 providers	Medication prescription	Implementing a genomic prescribing system that initiates an alert when a medication with pharmacogenomics information is prescribed to an enrolled patient and links genomic test results to the patient's record	Adoption of pharmacogenomic information.
Design and rationale of GUARDD-US: A pragmatic, randomized trial of genetic testing for APOL1 and pharmacogenomic predictors of antihypertensive efficacy in patients with hypertension [ 24 ]	Genetic screening, PGx	3,423 patients self-identified as African American/Black/Afro-Caribbean/Afro-Latino	Genetic based ordering system, return of genetic results system	CDS was implemented to perform APOL1 genotyping and returning the results to participants and providers via CDS system to encourage cardiovascular disease screening and select the right antihypertensive that led to changes in systolic blood pressure and diagnosing cardiovascular disease	Controlled systolic blood pressure
Implementing a Pharmacogenomic-driven Algorithm to Guide Antiplatelet Therapy among Caribbean Hispanics: A non-randomized prospective cohort study [ 18 ]	PGx	300 patients: 150 cases and 150 controls	Medication prescription	Providing actionable recommendations for optimal anti-platelet therapy to reduce the occurrence of major adverse cardiovascular and cerebrovascular events [MACCEs] among Caribbean Hispanic patients on clopidogrel	Major adverse cardiovascular and cerebrovascular events
Implementing Pharmacogenetic Testing in Gastrointestinal Cancers (IMPACT-GI): Study Protocol for a Pragmatic Implementation Trial for Establishing DPYD and UGT1A1 Screening to Guide Chemotherapy Dosing [ 19 ]	PGx	300 participants to be enrolled	Medication prescription	Assessing the feasibility of DPYD and UGT1A1 pharmacogenetic testing to guide fluoropyrimidine and irinotecan dosing in patients with GI malignancies, where the effectiveness is measured using patient-reported outcome (PRO), and quality of life (QoL) measures	Less adverse medication events, dose intensity
Prescriber Adoption of SLCO1B1 Genotype-Guided Simvastatin Clinical Decision Support in a Clinical Pharmacogenetics Program [ 20 ]	PGx	1,639 patients	Medication prescription	Assessing the implementation of Genotype-Guided CDS to guide simvastatin prescription for patients who had intermediate function OATP1B1 phenotype and had a poor function phenotype	Increased adoption of pharmacogenetics into routine practice when presenting test results clearly
Veterans Health Administration: Implementation of pharmacogenomic clinical decision support with statin medications and the SLCO1B1 gene as an exemplar [ 21 ]	PGx	480,102 patients	Medication prescription and management	Alerting patients and providers of actionable drug-gene interactions to reduce the risk of adverse drug reactions	PGx adoption, Adjusted Statin intensity
Clinical decision support with a comprehensive in-EHR patient tracking system improves genetic testing follow up [ 26 ]	Genetic screening/ Clinical Referral	768 patients	Genetic test ordering system	Implementing tracking system to improve genetic testing follow-up	Increasing documentation of follow-up recommendations after genetic testing
Linking Technology to Address the Social and Medical Determinants of Health for Safe Medicines Use [ 22 ]	Disease risk		Medication management	Evidence based at-home medication review and risk assessment tool that includes patients' psychological, functional need and home safety assessment	Lower number of drugs, reduce medication-induced harm
Does Clinical Decision Support Increase Appropriate Medication Prescribing for Cardiovascular Risk Reduction?	Disease risk	29,771 patients	Medication prescription	CDS identifies encounters with uncontrolled cardiovascular disease risk and recommends medications ordered from greatest to least benefit toward overall cardiovascular disease risk reduction	Adoption rate calculated from new evidence based EHR prescriptions for blood pressure, glucose, lipid, or tobacco control medications
Pyramidal Decision Support Framework Leverages Subspecialty Expertise across Enterprise to Achieve Superior Cancer Outcomes and Personalized, Precision Care Plans [ 27 ]	Disease Risk/genetic screening	In 2021: 56,143 unique patients where 8,807 were new patients	Alert system, treatment planning	1. Building four-tiered pyramidical decision support framework to provide precise treatment plan for cancer patients using clinical evidence and genes. 2. Creating CDS tool to alert providers when oncology patients who have inherited increased cancer risk might benefit from genetic assessment	5- and 10-year survival outcomes, addition of clinical trials, validating Clinical Pathway recommendations
Enhancing an AI-Empowered Periodontal CDSS and Comparing with Traditional Perio-risk Assessment Tools [ 28 ]	Risk disease	20 patients	Disease identification and evaluation	CDS system collects data, applies the model that calculates patient's periodontal disease risk, and generates visualizations for the web server's graphical user interface	Identifying Periodontal disease using risk score
Methods for development and application of data standards in an ontology-driven information model for measuring, managing, and computing social determinants of health for individuals, households, and communities evaluated through an example of asthma [ 29 ]	Social Determinant of Health		Disease identification and evaluation	Expanding terminologies and coding to manage and capture SDoH information and support its integration in the EHR. Evaluating the integration of environmental SDOH focusing on air quality	No outcome was measured
Development of an Electronic Health Record–Based Clinical Decision Support Tool for Patients With Lynch Syndrome [ 30 ]	Clinical Referral		Alert system for Guideline recommendation	Building CDS for Lynch Syndrome genomic indicator to recommend Colonic surveillance and annual genetic visit	Increase in adherence to cancer risk management such as colonic surveillance
Can Automated Alerts in the Electronic Health Record Encourage Referrals for Genetic Counseling and Testing Among Patients at High Risk for Hereditary Cancer Syndromes?[ 31 ]	Clinical Referral	Among 15,821 that triggered alert, 2,350 patients completed questionnaire, 357 patients triggering referral BP, 176 referrals were generated	Alert system	Creating CDS to alert providers when oncology patients who have inherited increased cancer risk might benefit from genetic assessment	Increase genetic assessment

Since most studies assessed the implementation of pharmacogenomics tools, their CDS tools focused on medication management and prescriptions to adjust medication dosage, find the right treatment, prevent adverse events, and identify drug-drug interactions [ 16 17 18 19 20 21 22 23 ]. Six studies enrolled less than 1,000 patients, while eight included more than 1,500 patients. Six studies implemented CDS systems to identify patients with cardiovascular disease, statin disease medication prescription and management, or cardiovascular genotyping [ 17 , 18 , 20 , 21 , 23 , 24 ]. Seven papers discussed educational efforts to train providers on the CDS tools.

All studies reported improvement in studied outcomes. Five studies reported higher adoption rates and referrals. Two studies reported lower drug-drug interactions and drug adverse events. Three studies reported better medication management by lowering the number of prescribed medications and providing the right dosage. Two papers reported lower significant events such as readmission or cardiovascular disease events. Three papers discussed alert fatigue and its influence on CDS implementation. Only one paper studied the implementation of SDoH in EHR.

## 4. Discussion

Our scoping review for the IMIA Yearbook showed a paucity of recently published CDS tools that have integrated novel social or biological data to support precision medicine. By expanding the breadth of precision medicine domains, our review shows that disease risks, SDoH, and systems to improve referral are starting to be implemented and evaluated. Such systems could advance the use of targeted therapies, increase cost-effectiveness, and improve patient outcomes such as changes in blood pressure and reduction in drug-drug interactions [ 24 , 32 33 34 ].

Most CDS systems that integrated genetic data used common genetic variants that have been already evaluated as reported by Smith *et al.* , within their literature review from 2021 [ 14 ]. However, new variants are being integrated into CDS, such as OPRM1 and CYP3A4, for opioid, antidepressant, and platelet inhibitor medication interactions. The scoping review showed that most CDS implementation mainly focused on cardiovascular diseases and drugs compared to other diseases such as cancer and diabetes that had fewer CDS implementations. Our review showed more studies focused on providing educational materials for providers and discussed the importance of offering training for CDS tools. However, only one study included educating patients and the community on pharmacogenomics testing; another recommended patient education on medication management [ 22 , 25 ]. As discussed below, having one main disease as the primary focus or selected variants can stem from the barrier of implementing CDS for new diseases due to translational barriers and an abundance of guidelines.

### 4.1. Barriers to Implementing Precision Medicine in CDS

The low level of implementation of precision medicine CDS tools that address common domains such as cardiovascular or commonly screened variants is due to multiple barriers. Those barriers can be broken down into the following categories: Data barriers, Knowledge barriers, data standard barriers, gaps in research translation, and infrastructure barriers.

#### 4.1.1. Data barriers

The data needed to implement precision medicine models are usually unavailable at the point of care due to incompleteness and limited early access to data implemented in precision medicine models. Genomic sequencing is generally performed after healthcare providers order genomic testing. However, some precision medicine knowledge and data barriers limit the implementation of precision medicine into CDS. The Genomic Medicine Working Group reported that incorporating multiple genes and related clinical knowledge is still a major issue in implementation [ 35 ]. Most diseases are caused or affected by multiple genes; hence, accurate risk assessment requires accessing all relevant genetic loci and relevant clinical factors [ 36 , 37 ]. Currently, CDS systems cannot combine the data from genomic and non-genomic data sources [ 17 , 38 ].

#### 4.1.2 Knowledge barriers

The need for genomic education and literacy can limit the adoption and implementation of precision medicine in CDS [ 39 ]. In a systematic review of barriers to implementing precision medicine in practice, Qureshi reported that lack of patients' and providers' knowledge in genetics, and limited experience, education, and training in pharmacogenomics are common knowledge barriers [ 40 , 41 ]. Incorporating precision medicine knowledge ( *e.g.* , pharmacogenomics, genotyping) into training, undergraduate or postgraduate education, or learning from collogues programs can increase awareness and adoption [ 22 , 40 , 41 ]. Obeng found that presenting genetic knowledge, through one hour training session, clearly and effectively can improve prescriber systems' implementation and adoption effectiveness [ 20 ].

#### 4.1.3. Data Standard Barriers

The lack of standards can be another barrier. Genomic sequencing is mainly performed by separate laboratories that do not provide the data in a computable standardized format and provide a subset of the genomics data, which can limit the potential of genomics analysis [ 36 , 37 ]. In addition, there is a lack of standardization on reporting variants with unknown significance, rare or private significance, where some laboratories do not report those variants due to concerns about quality control and assurance [ 42 ]. Welch *et al.* included genomic data standards as one of the pillars of using whole genome sequence in CDS [ 37 ]. The absence of standard reporting of pharmacogenomic test results and standard representation of genomic data can limit the implementation of pharmacogenomics tools in one healthcare institution and affect its scalability when deploying the CDS tools across different institutions [ 35 , 43 ]. In SDoH, only one manuscript described the implementation of SDoH. It concluded that a standard SDoH ontology plays a crucial role in integrating SDoH data in the EHR and building precise CDS tools [ 29 ]. Developing unified standards to store and exchange genetics and other precision medicine data is a vital step to scale the integration effort, especially in fields still in their infancy regarding data standardization, such as pharmacogenomics [ 44 ].

#### 4.1.4. Gaps in Research Translation

Translating and adopting precision medicine models such as machine learning and genotyping models is challenging [ 45 , 46 ]. Models are trained and evaluated in research environments on data preprocessed. The differences between the real world and the research environment can slow down the adoption of the model [ 43 ]. For instance, the requirements of genomic arrays and essays in clinical laboratories differ in research laboratories, which can delay the implementation in clinical workflow [ 47 ].

Ethical implications are a barrier to translating research to the real world. Health inequity, such as having fewer recommendations for genetic or lower germline genetic testing and disease detection for non-white patients, can lead to biased models hindering or skewing translation into clinical settings [ 26 , 30 , 48 ]. Varughese *et al.* , reported that some oncologists expressed ethical concerns with a randomized trial design [ 19 ]. In addition, some studies are conducted in single-institution settings, which limits the generalizability of the model performance [ 28 ]. Ensuring successful translation may require non-randomized trials, adjusting for bias, performing multi-institutional models and knowledge representation and considering the ethical implications of applying precision models.

#### 4.1.5. Infrastructure

Few EHR institutions can allocate sufficient local effort to create a comprehensive strategy to integrate genomics into the EHR. While vendors have started to address the gap, it is still early for widespread adoption [ 49 ]. The current infrastructure must be better equipped to integrate multi-omics data such as genomics, environmental, or biometric data. Zorn reported that current EHR systems do not support capturing detailed clinical information required for genetic assessment [ 31 ]. Current EHR systems may require the proper infrastructure or standard data containers to store genetic or environmental data, especially when incorporating multi-omics data and accounting for security requirements [ 37 , 42 , 50 ]. Moreover, patients usually encounter technical issues, whether during the consent process for genetic testing, being enrolled in trials, or providing SDoH or environmental data, which require additional infrastructure resources [ 43 , 51 ].

Out of 54 papers included for full-text review, 35 studies investigated the usage of precision medicine in CDS and discussed the possible usages but lacked the implementation stage. Other reviews and survey studies that examined the implementation of precision medicine in CDS found fewer implemented CDS tools. In a review that focused on improving patient outcomes using pharmacogenetic interventions implemented in EHR, the authors screened 10,725 papers and included 12 papers in the review [ 52 ].

Our scoping review included studies that implemented other aspects of precision medicine, such as SDoH and individualized risk score. The review has several limitations: first, the strategy may have missed relevant studies due to the diversity of keywords that may be attached to articles. Second, we limited our search to studies in PubMed with full-text access.

## 5. Conclusion

The implementation of precision medicine in CDS improves the treatment and diagnosis process. Pharmacogenomics is still the main precision medicine implemented in EHR with the target to prescribe the right drug. The number of implemented precision medicine CDS is still low. Knowledge barriers and infrastructure challenges can slow down the implementation. However, our scoping review demonstrates that additional precision medicine domains, such as disease risk, clinical referrals, and SDoH, are motivating the creation of CDS. Future reviews should examine the implementation trends in precision medicine, particularly beyond the use of genomics data.
